# Subacute Microperforation of Right Atrial Pacemaker Lead Presenting with Shock due to Cardiac Tamponade: A Case Report

**DOI:** 10.5811/cpcem.63991

**Published:** 2026-09-20

**Authors:** Alexander Zirulnik, Briton Bennett, Siham Almousa

**Affiliations:** Wake Forest University School of Medicine, Department of Emergency Medicine, Winston-Salem, North Carolina

**Keywords:** cardiac tamponade, pacemaker lead perforation, point-of-care ultrasound, pericardial effusion, obstructive shock

## Abstract

**Introduction:**

Delayed cardiac tamponade is a rare but life-threatening complication following permanent pacemaker placement and may present with non-specific symptoms.

**Case Report:**

We report the case of a 66-year-old woman on oral anticoagulation who presented with generalized weakness, nausea, and profound hypotension four days after dual-chamber pacemaker placement. Initial evaluation demonstrated atrial fibrillation with rapid ventricular response, a normal chest radiographic findings, prerenal azotemia, and no evidence of device malfunction on interrogation. Point-of-care ultrasound (POCUS) performed in the emergency department revealed a large pericardial effusion with right atrial and right ventricular collapse and a plethoric inferior vena cava, consistent with cardiac tamponade. Emergent pericardiocentesis yielded 400 mL of sanguinous pericardial fluid with rapid hemodynamic improvement. Subsequent imaging revealed a 2-mm protrusion of the right atrial lead into the myocardium, which was suspected to be the source of the effusion, but pacemaker revision was not necessary.

**Conclusion:**

This case highlights the limitations of routine diagnostic testing following permanent pacemaker placement and emphasizes the importance of early POCUS in patients with atypical presentations, particularly in the setting of anticoagulation.

## INTRODUCTION

Permanent pacemaker placement is a common procedure with generally low complication rates. However, serious complications such as cardiac perforation and pericardial effusion leading to cardiac tamponade can occur, either immediately or in a delayed fashion. Delayed cardiac tamponade can be particularly challenging to diagnose due to nonspecific symptoms and potentially reassuring initial diagnostic studies, including normal chest radiography and device interrogation.

Emergency physicians must maintain a high index of suspicion for postprocedural complications, especially in anticoagulated patients. Point-of-care ultrasound (POCUS) has emerged as a critical tool in the rapid identification of pericardial effusion and tamponade physiology in unstable patients.

## CASE REPORT

A 66-year-old woman with a history of nonobstructive coronary artery disease, Hodgkin lymphoma, breast cancer status postbilateral mastectomy, heart failure with recovered ejection fraction (50%), and persistent atrial fibrillation/flutter on rivaroxaban presented to the emergency department (ED). with generalized weakness and hypotension.

Three days prior to presentation, she underwent dual-chamber permanent pacemaker placement and was discharged in stable condition. She reported feeling well immediately following discharge until earlier in the day of presentation when she developed progressive generalized weakness and fatigue, accompanied by nausea and one episode of vomiting. She noted home blood pressure readings as low as 60/30 mm Hg, but denied chest pain, dyspnea, syncope, or lower extremity swelling.

On arrival, she appeared toxic and in extremis. Her triage vital signs were notable for hypotension (61/44 mm Hg) and a heart rate of 136 beats per minute. Physical exam was notable for irregular tachycardia, clear lung sounds, pale skin, diaphoresis, healing procedural sites without evidence of infection, and no clear signs of volume overload. Given her hypotension and tachycardia, she received a 1 L bolus of crystalloid resuscitation but remained persistently hypotensive. Subsequently, POCUS demonstrated newly reduced ejection fraction (estimated between 30% and 50%; normal ejection fraction ≥ 50%), a large pericardial effusion with right atrial systolic collapse, right ventricular diastolic collapse, and a plethoric inferior vena cava with minimal respiratory variation, consistent with cardiac tamponade (Images 1 and 2). Cardiology was consulted.

Laboratory studies revealed the following: lactate, 2.7 mmol/L (reference range, 0.5–2.2 mmol/L); white blood cell count, 11.5 × 10^3^/μL (4.4–11.0 × 10^3^/μL); and creatinine, 1.22 mg/dL ( 0.70–1.30 mg/dL), increased from 0.70 mg/dL four days prior. Electrocardiogram demonstrated atrial fibrillation with rapid ventricular response. Chest radiograph showed no acute cardiopulmonary abnormalities. Device interrogation in the ED demonstrated no evidence of dysfunction. The patient’s vitals marginally improved after fluid support, and after cardiology consultation, she was admitted to the cardiovascular intensive care unit (ICU) to undergo urgent pericardiocentesis.

Pericardiocentesis was performed with removal of 400 mL of sanguinous pericardial fluid and hemodynamics improved significantly. A pericardial drain was placed, and she returned to the cardiovascular ICU.

*CPC-EM Capsule*

What do we already know about this clinical entity?

*Delayed cardiac tamponade after pacemaker placement is rare, may present atypically, and can be difficult to diagnose with routine testing.*

What makes this presentation of disease reportable?

*Cardiac tamponade developed days after pacemaker placement despite normal chest radiography and device interrogation; rapid point-of-care ultrasound (POCUS) established the diagnosis.*

What is the major learning point?

*Early POCUS is essential in unstable patients after recent pacemaker implantation to identify cardiac tamponade.*

How might this improve emergency medicine practice?

*Routine early POCUS in hypotensive patients after pacemaker implantation can expedite diagnosis of tamponade and lifesaving intervention.*

She developed pleuritic chest pain immediately following the procedure and was started on nonsteroidal anti-inflammatory drug treatment for pericarditis. Repeat transthoracic echo on postoperative day 1 demonstrated a reduced ejection fraction of 30% without recurrence of pericardial effusion.

Computed tomography of the chest on postoperative day 3 revealed approximately 2 mm protrusion of the right atrial lead into the myocardium, and in the setting of the patient’s use of a direct-acting oral anticoagulant, this was considered to be the most likely culprit for her pericardial effusion. The electrophysiology consultation determined that lead revision was not indicated. Maintaining the current position may have tamponaded the perforation, and revision could have carried a higher risk of complication. Furthermore, serial device interrogation demonstrated no device malfunction.

Anticoagulation was resumed after stabilization without reaccumulation of the effusion. In total, approximately 1.2 L of fluid was drained before removal on day 4 of her admission. The patient was ultimately discharged in stable condition.

## DISCUSSION

Delayed cardiac tamponade following permanent pacemaker placement is an uncommon but well-described complication; however, most are recognized early. Cardiac tamponade causing hemodynamic instability secondary to acute perforation beyond 72 hours accounts for a small minority of presentations.^1^ The fact that the patient was on a direct-acting oral anticoagulant likely contributed to both the timing of her presentation and the severity of her tamponade, and patients on direct oral anticoagulants may be at increased risk of hemorrhagic pericardial effusion in the setting of microperforation.^2^ In the largest contemporary series, anticoagulation status was an independent predictor of tamponade, and anticoagulated patients had approximately a 22-fold increased risk of developing tamponade when perforation occurred.^3^

The patient’s clinical presentation was also atypical. Rather than classic features such as chest pain, which is the most common presenting symptom, she presented with generalized weakness, nausea, and profound hypotension.^3^ Such nonspecific symptoms may delay recognition, particularly in patients with competing diagnoses such as arrhythmia or hypovolemia.^4^

This case highlights the limitations of commonly used diagnostic tools. Chest radiography may not detect lead perforation or pericardial effusion, and normal device interrogation does not exclude mechanical complications, such as microperforation.^5^ In this patient, both studies were nondiagnostic despite the presence of life-threatening tamponade physiology. However, POCUS played a pivotal role in diagnosis and allowed for rapid identification of tamponade physiology and expedited definitive management. Prior studies have demonstrated that POCUS significantly improves time to diagnosis in undifferentiated hypotension and is highly sensitive for detecting pericardial effusion.^6^ Notably, it was not until several days into her hospitalization when microperforation was uncovered.

We believe this reinforces the importance of early POCUS in unstable patients and highlights the ability of emergency physicians to quickly and definitively expose life-threats even when traditional testing may be nonconclusive, nondiagnostic, or superseded by the need to stabilize a critically ill patient.

## CONCLUSION

Delayed cardiac tamponade following pacemaker placement can present with nonspecific symptoms and misleadingly normal initial diagnostic studies. Clinicians should maintain a high index of suspicion in recently instrumented and anticoagulated patients presenting with hypotension. Point-of-care ultrasound is a critical tool for early diagnosis and may be life-saving in these scenarios.

## Figures and Tables

**Image 1:**
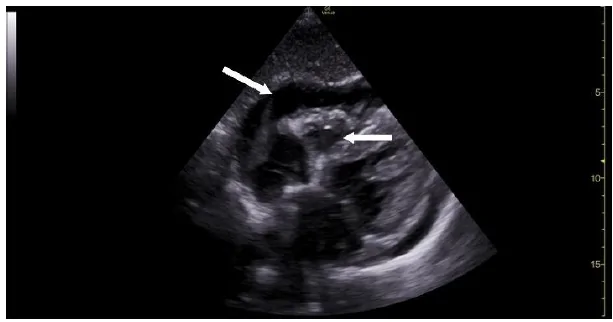
Point-of-care ultrasound using a subxiphoid cardiac view demonstrates a large pericardial effusion (left arrow) with right ventricular diastolic collapse (right arrow). These findings were consistent with cardiac tamponade physiology.

**Image 2:**
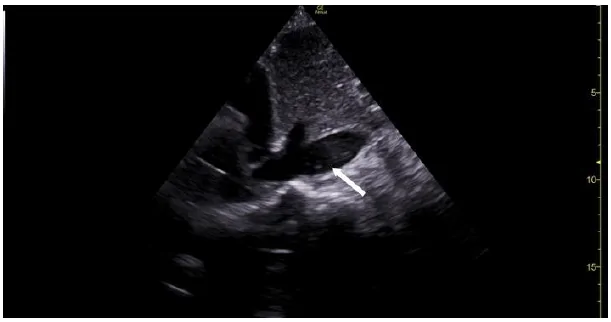
Point-of-care ultrasound of the inferior vena cava (IVC) (white arrow) demonstrates a plethoric, dilated IVC with minimal respiratory variation, consistent with elevated right atrial pressure in the setting of cardiac tamponade.
